# The Characterization of a Chitosan Coating Enriched with Bamboo-Leaf Flavonoids and Its Effect on Postharvest Preservation of Three Horticultural Products

**DOI:** 10.3390/foods14132364

**Published:** 2025-07-03

**Authors:** Haoran Wu, Feng Tang, Xi Yao

**Affiliations:** Key Laboratory of National Forestry and Grassland Administration on Bamboo & Rattan Science and Technology, International Center for Bamboo & Rattan, Beijing 100102, China; haoranwu0310@163.com (H.W.); fengtang@icbr.ac.cn (F.T.)

**Keywords:** chitosan coating, antioxidant activity, bamboo-leaf flavonoids, *Fragaria × ananassa* (strawberry), *Vaccinium corymbosum* (blueberry), *Phyllostachys praecox f. prevemalis* (bamboo shoots)

## Abstract

Chitosan coatings have been demonstrated to be a highly effective and safe approach to extending the shelf life of food. This study, for the first time, evaluates the effectiveness of bamboo-leaf flavonoids (BLFs) added to a chitosan coating to delay the spoilage of strawberries, blueberries, and bamboo shoots. The addition of BLFs improved the tensile strength of the coatings. Chitosan coating incorporated with 0.1% BLFs had the highest tensile strength (36.38 ± 2.69 MPa). BLFs conferred antioxidant properties to chitosan coatings as determined by DPPH radical scavenging activity. Key quality parameters were measured over the storage period of strawberries, blueberries, and bamboo shoots. The coating significantly affected the impact of storage time on some variables. Chitosan/BLF coatings were particularly effective in limiting changes over time in weight loss, spoilage percentage, and vitamin C content (strawberries and blueberries), as well as crude fiber content (bamboo shoots), although their effect on titratable acid, soluble solids, and soluble protein content was less pronounced. The chitosan/BLFs composite coating demonstrated superior efficacy over pure chitosan in delaying spoilage. In conclusion, the chitosan/BLF coating could be useful for maintaining the quality of strawberries, blueberries, and bamboo shoots.

## 1. Introduction

Strawberries (*Fragaria × ananassa*), blueberries (*Vaccinium corymbosum*), and bamboo shoots (*Phyllostachys praecox f. prevemalis*) are highly perishable crops with significant nutritional and economic value. Strawberries and blueberries are rich in vitamins, polyphenols, and antioxidants, but they are prone to rapid spoilage during postharvest storage. This deterioration is driven by their high moisture content, microbial growth, and oxidative degradation (caused by free radical formation), leading to color changes, nutrient loss, and texture degradation) [1,2,3,4,5,6]. Bamboo shoots, a popular vegetable in Asia, are valued for their dietary fiber and bioactive compounds but undergo rapid deterioration through enzymatic browning and lignification [7,8,9]. Extending the shelf life of these produce items is critical to reducing postharvest losses, ensuring food security, and maintaining nutritional quality.

Chitosan, a biodegradable polysaccharide derived from chitin, has emerged as a promising edible coating material due to its film-forming ability, antimicrobial properties, and compatibility with bioactive additives [10,11,12,13]. Chitosan coatings act as semi-permeable barriers, reducing respiration rate, water loss, and microbial contamination in fruits and vegetables [14,15]. The mechanical properties of coatings play a pivotal role in determining their effectiveness for preservation. Although pure chitosan coatings can form films, their insufficient mechanical strength, particularly in terms of tensile ductility and fracture resistance, can lead to coating damage during storage and transportation [16,17,18]. Such damage, often caused by external forces like physical compression and vibration, compromises the coating’s barrier against water loss, microbial invasion, and oxidation [17,19]. Furthermore, incorporating plant extracts into chitosan films is a common strategy to enhance their biological properties, primarily antimicrobial and antioxidant properties [20,21].

Bamboo-leaf flavonoids (BLFs), a class of natural polyphenols, exhibit strong antioxidant and antimicrobial activities [22,23] and have been included in China’s “National Food Safety Standard—Standard for the Use of Food Additives” (GB 2760-2024) [24]. Incorporating BLFs into chitosan coatings could potentially enhance their functional properties synergistically. Since 2008, there has been growing interest in grafting phenolic compounds onto chitosan through copolymerization [25,26,27]. Phenolic compounds can be classified into five main categories based on their structure: phenolic acids, flavonoids, tannins, stilbenes, and lignans [28]. Previous studies have demonstrated that phenolic compounds, such as green tea extract or grape seed extract, improve the mechanical strength and antioxidant capacity of biopolymer films by forming hydrogen bonds with the polymer matrix [29,30,31,32]. While the role of chitosan/bamboo-leaf flavonoids/nano-metal oxide composite films in rapeseed oil antioxidation has been studied [33], the application of BLFs in chitosan-based coatings to exploit their potential antimicrobial, antioxidant, and synergistic functional properties remains largely unexplored.

This study aims to develop a chitosan/BLFs composite coating and evaluate its efficacy in prolonging the shelf life of strawberries, blueberries, and bamboo shoots. The specific objectives include (1) optimizing the BLF concentration to enhance the mechanical and antioxidant properties of chitosan films and (2) assessing the impact of chitosan/BLF coatings on key quality parameters (e.g., weight loss, vitamin C retention) during storage. We hypothesize that BLFs will strengthen the chitosan matrix through intermolecular interactions while conferring antioxidant activity, thereby delaying oxidative and microbial spoilage in the coated produce.

## 2. Materials and Methods

### 2.1. Materials

Strawberries (*Fragaria × ananassa* ‘Red Face’, eighth-maturity stage) and blueberries (*Vaccinium corymbosum*, fully ripe) were sourced from a local market. Bamboo shoots (*Phyllostachys praecox f. prevemalis*, ≤48 h postharvest, tender apex, no lignification) were picked from Jianou City, Fujian Province, China. Chitosan powder was purchased from Sinopharm Chemical Reagent Co., Ltd. (Beijing, China). Bamboo-leaf flavonoids (EOB-SS01, flavonoid content: isoorientin 3.224%; orientin 0.727%; vitexin 0.109%; isovitexin 0.321%) were obtained from Shengshi Biotechnology Co., Ltd. (Anji, China). 1,1-diphenyl-2-picrylhydrazyl (DPPH) and sodium hydroxide standard titration solution (0.1 M) were purchased from Macklin Biochemical Technology Co., Ltd. (Shanghai, China). 2,6-dichloroindophenol (DPIP) was purchased from Yien Chemical Technology Co., Ltd. (Shanghai, China). Coomassie Brilliant blue G-250 (C.I. 42655) was purchased from Aladdin Biochemical Technology Co., Ltd. (Shanghai, China). Other reagents were of analytical grade.

### 2.2. Preparation of Composite Films

Film-forming mixtures containing 0.5% (*w*/*v*) chitosan and 0.15% (*w*/*v*) glycerol dissolved in 1% (*w*/*v*) acetic acid were prepared. The mixtures were homogenized for 3 h at room temperature using magnetic stirrers. Subsequently, BLFs were incorporated at a concentration of 0.025%, 0.05%, 0.075%, 0.1%, and 0.2% (*w*/*v*) (Table 1), under stirring for 30 min at 50 °C. The obtained mixtures were homogenized for 5 min using a high-shear homogenizer (FA25, Fluko, Berlin, Germany) at 16,000 rpm and then degassed using an ultrasonic cleaner for 50 min. The films were prepared by casting 100 mL of solution onto Teflon plates (10 cm × 10 cm) and drying at 60 °C in a ventilated oven to obtain films of uniform thickness. The dried films were peeled from the casting surface. Film characteristics were determined after all sample films were preconditioned in a constant temperature humidity chamber (KBF 115, Binder, Neckarsulm, Germany) set at 25 °C with 43% RH for 24 h.

### 2.3. Mechanical Properties of Coatings

Tensile strength (TS, MPa) and elongation at break (EAB, %) were determined using an electronic universal testing machine (CMT6503, MTS Systems Corporation, Eden Prairie, MN, USA) according to ISO Standard Method 527-3:2018 [34]. Films were cut into strips 15 mm wide and 80 mm long. The initial grip distance and crosshead speed were 50 mm and 20 mm/min, respectively. At least five replicates of each film were tested.

### 2.4. DPPH Radical Scavenging Assay

The DPPH radical scavenging activity of samples was measured using a multilabel reader (Victor X3, Perkin Elmer, Waltham, MA, USA) according to the method described by Li et al. [35] with a slight modification. Briefly, 2.0 mL aliquots of film solution were combined with 2.0 mL DPPH ethanol solution (50 mg/L), the mixture was incubated at room temperature in the dark for 15 min, and the absorption was measured at 517 nm (A_1_). Control measurements were also taken: 2.0 mL ethanol mixed with 2.0 mL of 50 mg/L DPPH ethanol solution (A_2_), and 2.0 mL of each film solution mixed with 2.0 mL ethanol (A_3_). The percentage of DPPH radical scavenging activity was calculated as follows:

(1) $$\mathrm{DPPH}\;\mathrm{scavenging}\;\mathrm{activity}\;\left(\%\right)=\left[1-\left(A_{1}-A_{3}\right)/\left(A_{2}-A_{3}\right)\right]\;\times \;100\%,$$

where A_1_, A_2_, and A_3_ are the absorbance at 517 nm of the DPPH solution with film solution, DPPH solution with ethanol, and film solution with ethanol, respectively. Three independent replicates were performed for each sample.

### 2.5. Application of Chitosan/BLFs in the Preservation of Strawberries, Blueberries, and Bamboo Shoots

The strawberries, blueberries, and bamboo shoots were randomly distributed into five groups before treatment. The produce was then dipped in the respective film solution (CS, CS/BLFs-50, CS/BLFs-75, and CS/BLFs-100) for 60 s to allow the chitosan to adhere uniformly to the surface. All samples were packaged in polyethylene (PE) plastic ziplock bags and stored at 25 ± 2 °C with a relative humidity (RH) of 55 ± 5%. Each treatment comprised 30 samples (10 bags × 3 units per bag), with three biological replicates. Strawberries were sampled and tested on days 0, 3, 4, 5, 6, 7, and 8. Blueberries were sampled and tested on days 0, 3, 5, 7, 8, 9, and 10. Bamboo shoots were sampled and tested on days 0, 3, 5, 6, 7, 8, and 9.

#### 2.5.1. Appearance Assessment of Strawberries, Blueberries, and Bamboo Shoots

Visual assessment included color uniformity, plumpness, and presence of lesions. Images were captured daily throughout the storage period.

#### 2.5.2. Weight Loss and Spoilage Percentage of Strawberries, Blueberries, and Bamboo Shoots

Weight loss percentage was calculated based on the weight of each sample before and after storage and expressed as the percentage weight loss compared to the initial weight. The spoilage percentage was calculated based on the proportion of fruits with mold spots or soft rot on their surface as follows:

(2) $$Spoilage\left(\%\right)=\frac{\sum N_{t}}{N_{0}},$$

where *N_t_* is the number of rotten fruits, and *N*_0_ is the original total number of fruits.

#### 2.5.3. Vitamin C Content of Strawberries, Blueberries, and Bamboo Shoots

Vitamin C (VC) content was measured according to the China National Standards GB/T 5009.86-2016 [36].

#### 2.5.4. Titratable Acid and Soluble Solid Content of Strawberries and Blueberries

Titratable acid (TA) content was measured according to the China National Standards GB/T 12456-2021 [37]. Soluble solid (TSS) content was measured using a refractometer (WAY 2S digital Abbe refractometer, Shanghai Precision Scientific Instrument Co., Ltd., Shanghai, China) according to the China Agricultural Industry Standards NY/T 2637-2014 [38]. Three independent replicates were performed for each sample.

#### 2.5.5. Soluble Protein Content of Bamboo Shoots

The soluble protein content was determined according to the method described by Bradford et al. [39] with appropriate modifications. Briefly, 2.0 g of the bamboo shoot sample was mixed with 5 mL of distilled water and thoroughly ground into a homogenate, followed by centrifugation. Then, 1 mL of the supernatant was mixed with 4 mL of Coomassie Brilliant Blue G-250 solution, and the absorbance was measured at 595 nm. Three independent replicates were performed for each sample.

#### 2.5.6. Crude Fiber Content of Bamboo Shoots

Crude fiber content was measured according to the Chinese National Standard GB/T 5009.10-2003 [40]. Briefly, 10 g of crushed bamboo shoot sample was treated with 1.25% H_2_SO_4_ to hydrolyze oligosaccharides, starch, pectin, and hemicelluloses. Subsequently, protein and fatty acid were eliminated by 1.25% NaOH, and the remaining crude fiber was extracted. The weight ratio of the dried crude fiber to the initial bamboo shoot sample was calculated as the crude fiber content (%). Three independent replicates were performed for each sample.

### 2.6. Statistical Analysis

Data are expressed as the mean ± standard deviation (SD), and error bars in figures represent SD. To determine differences between uncoated, chitosan-coated, and chitosan/BLFs-coated fruits (strawberries, blueberries, and bamboo shoots), two-way ANOVA followed by Tukey‘s multiple comparisons test was used. Differences at *p* < 0.05 were considered significant. All analyses were performed using the GraphPad software package (version 10.2.3 (347), GraphPad Software, Inc., San Diego, CA, USA).

## 3. Results and Discussions

### 3.1. Optimization of Chitosan/BLFs Composite Films

#### 3.1.1. Mechanical Properties

**Figure 1:**
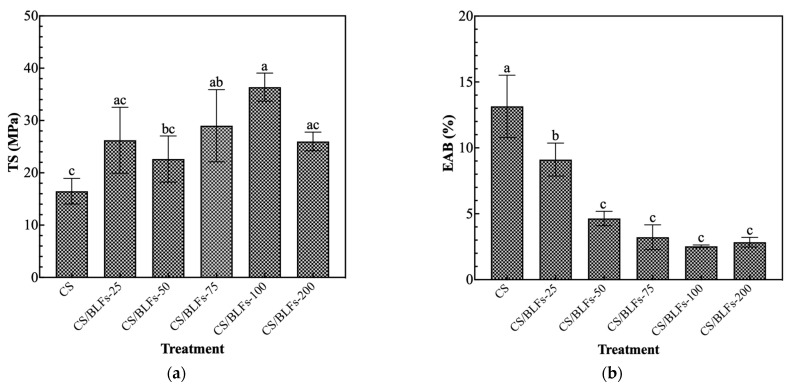
The effect of the addition of BLFs on mechanical properties. (**a**) tensile strength; (**b**) elongation at break. Bars with different letters indicate significantly different values (*p* < 0.05).

The inadequate mechanical properties of pure chitosan packaging can compromise its preservation performance, an issue that can be improved by incorporating supplementary components [41]. The addition of BLFs significantly impacted the mechanical properties of the chitosan coating, as evidenced by changes in tensile strength (TS) and elongation at break (EAB). As shown in Figure 1a, all chitosan films with added BLFs had higher tensile strength than pure chitosan films. The TS value of pure chitosan film (CS) was 16.48 ± 2.45 MPa. Although chitosan films with 0.025% BLFs (CS/BLFs-25) showed a higher value, the difference compared to pure chitosan was not significant. Increasing the BLF concentration to 0.1% (CS/BLFs-100) led to a peak TS of 36.38 ± 2.69 MPa, indicating an optimal concentration for enhancing strength. Phenolic compounds contain multiple OH groups that can form hydrogen bonds with chitosan [42]; this interaction is likely responsible for the increase in the tensile strength upon adding BLFs. This finding aligns with Siripatrawan and Harte’s [43] report of enhanced tensile strength in chitosan films incorporated with green tea extract, attributed to interactions between the chitosan matrix and polyphenolic compounds. Similarly, Sivarooban, Hettiarachchy, and Johnson [44] found that grape seed extract significantly boosted the tensile strength of soy protein isolate films. At a concentration of 0.2% BLFs (CS/BLFs-200), TS decreased, likely due to agglomeration of BLF particles acting as stress concentrators. Regarding the elongation at break (Figure 1b), a clear decreasing trend was noted with increasing BLF addition. EAB values decreased steadily from the control (0% BLFs) to the highest tested concentration (0.2% BLFs), suggesting that BLFs make the material more brittle by restricting the chain mobility of the chitosan polymer. These results highlight the complex role of BLFs in modifying the mechanical behavior of the chitosan coating.

#### 3.1.2. DPPH Radical Scavenging Ability

Antioxidant properties, especially radical scavenging activities, are crucial due to the damaging effects of free radicals in foods and biological systems [45]. The DPPH radical assay is widely used to evaluate the free radical scavenging ability and antioxidant activity of compounds [46].

The antioxidant capacity of chitosan/BLFs composite films was evaluated via DPPH radical scavenging assays, demonstrating a significant enhancement in activity with increasing BLF concentration. At a low BLF concentration of 0.025% (0.25 mg/mL), the scavenging rate was 44.22 ± 4.76%. Increasing the concentration to 0.05% (0.5 mg/mL) significantly elevated the rate to 63.38 ± 2.66% (*p* < 0.05). The scavenging activity peaked at 73.01 ± 1.65% with 0.1% BLFs (1.0 mg/mL), with only a marginal increase observed at 0.2% (2.0 mg/mL), indicating a saturation effect at higher concentrations.

This concentration-dependent trend confirms BLFs as a potent natural antioxidant. Numerous studies have confirmed the excellent antioxidant capacity of BLFs [22,23,47,48,49]. The phenolic hydroxyl groups in BLFs donate hydrogen atoms to DPPH radicals, neutralizing their oxidative activity. This mechanism aligns with previous findings that flavonoids with multiple aromatic hydroxyl groups exhibit superior radical scavenging capacity [50].

### 3.2. Application of Chitosan/BLFs in the Preservation of Strawberries and Blueberries

#### 3.2.1. Appearance of Strawberries and Blueberries

**Figure 2:**
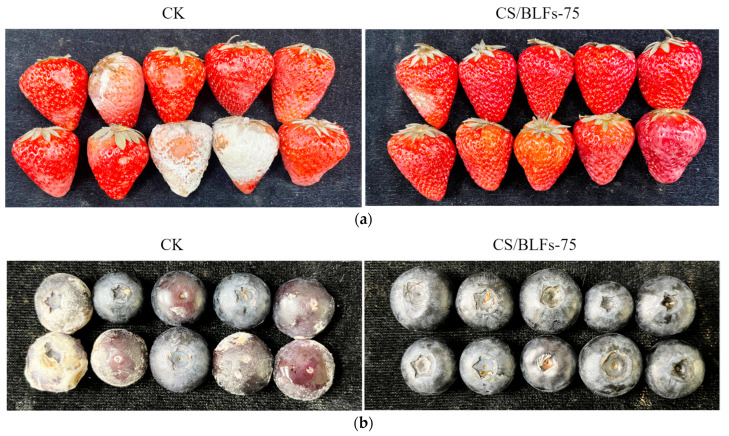
The appearance of (**a**) strawberries on day 8 and (**b**) blueberries on day 10.

The freshness of strawberries and blueberries can be visually assessed through appearance changes. The visual appearance during storage was significantly influenced by chitosan/BLF coatings, as shown in Figure 2. For strawberries, by day 4, the control group (CK) showed slight color fading at the apex and subtle loss of turgidity, while the chitosan-coated (CS) and CS/BLF-treated groups maintained better color saturation and surface integrity. Notably, the CS/BLFs-75 group displayed minimal changes, with vibrant red color and no visible water loss symptoms. By day 8, control groups showed severe wrinkling, softening, and darkening, while the CS/BLFs-75 group maintained relatively good firmness. For blueberries, by day 10, the control group (CK) exhibited shrinkage, darkened color, and cracked epidermis. In contrast, chitosan/BLF-coated groups maintained better appearance integrity (uniform color, no obvious damage or mold spots): the CS group showed deeper color and slight shrinkage, while CS/BLFs-50, CS/BLFs-75, and CS/BLFs-100 groups maintained a good appearance. The CS/BLFs-75 group retained a smooth surface and vibrant color.

These visual observations correlated with quantitative variables (e.g., spoilage percentage, weight loss percentage) measured subsequently, demonstrating that incorporating BLFs into chitosan coatings effectively prolonged the visual shelf life of strawberries and blueberries by inhibiting microbial growth [13], reducing water loss [51], and preventing oxidative discoloration [52]. The optimal BLF concentration (0.075%) appeared to enhance the coating’s barrier properties, providing the most pronounced preservation of fruit appearance.

#### 3.2.2. Weight Loss and Spoilage Percentage of Strawberries and Blueberries

**Figure 3:**
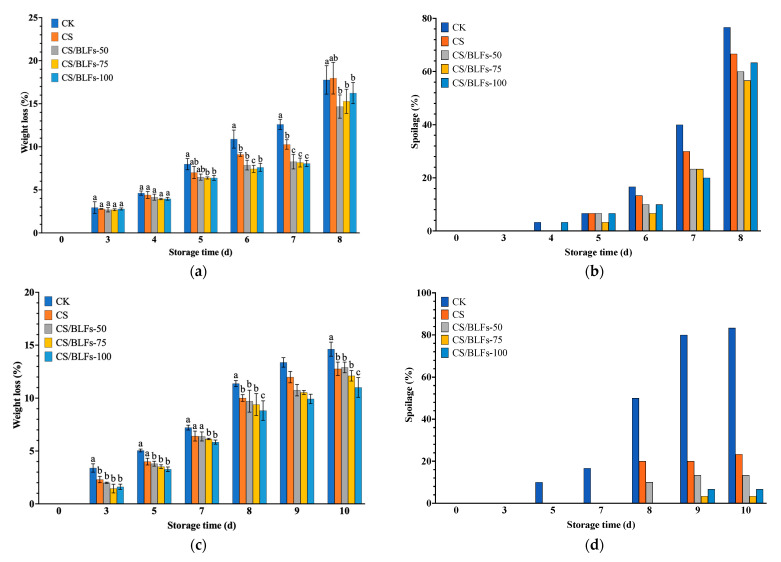
Weight loss and spoilage percentage of strawberries (**a**,**c**) and blueberries (**b**,**d**) stored at 25 °C for 8 and 10 days, respectively. Bars indicate the standard error of the mean. Columns labelled with different letters indicate significant differences with *p* < 0.05.

Chitosan coatings form a semi-permeable film on the fruit surface, limiting respiration and transpiration [53]. As depicted in Figure 3, weight loss percentage during storage was significantly affected by chitosan/BLF coatings for both strawberries (Figure 3a) and blueberries (Figure 3c). For strawberries, weight loss in the control group (CK) increased steadily, reaching 17.77% by day 8. The chitosan-coated (CS) group exhibited lower weight loss than CK in the early storage period. Among the CS/BLF groups, weight loss was further reduced. The CS/BLFs-50 group showed the lowest weight loss at most time points, with only about 14.67% loss by day 8. Blueberries’ weight loss showed a highly similar trend. The control group (CK) showed a steady increase, reaching 14.63% on day 10, significantly higher than all coated groups (*p* < 0.001, Figure 3c). Chitosan coating (CS) reduced weight loss to 12.78% on day 10, while CS/BLF treatments further mitigated water loss: CS/BLFs-100 exhibited the lowest weight loss (11.02% on day 10). At room temperature (25 °C), accelerated moisture evaporation led to higher weight loss in the control group (17.77% for strawberries and 14.63% for blueberries). In contrast, the chitosan/BLF coatings effectively reduced this loss by enhancing the moisture barrier. These results indicate that adding BLFs to chitosan coating enhances its ability to reduce water loss in strawberries and blueberries. The coating likely formed a more effective barrier through hydrogen bonding and electrostatic interaction, restricting water evaporation from the fruit surface [54,55].

Regarding spoilage percentage, for strawberries (Figure 3b), the control group (CK) started showing signs of spoilage as early as day 4, with a rapid increase thereafter. By day 8, the CK group showed extensive decay (>50% surface coverage), reaching nearly 76.67%. The CS group also showed a certain degree of decay, whereas the CS/BLFs-75 group retained approximately 80% of its original appearance, with mold development limited to isolated spots (spoilage percentage ~56.67%), demonstrating remarkable inhibition. For blueberries (Figure 3d), the spoilage percentage increased rapidly in the CK group, reaching 50% by day 8 and 83.33% by day 10. CS coating delayed spoilage onset (23.33% on day 10), while CS/BLFs-75 and CS/BLFs-100 significantly suppressed microbial growth, achieving spoilage percentages of only 3.33% and 6.67%, respectively, on day 10.

Chitosan has demonstrated potent antimicrobial activity against a broad spectrum of pathogenic and spoilage microorganisms, including fungi, Gram-positive, and Gram-negative bacteria [13,56]. The addition of BLFs enhances chitosan’s antimicrobial spectrum and potency, similar to other polyphenolic extracts. Studies show that polyphenols from green tea extracts [57], pomegranate peel extract [58], and apple peel extract [59] improve chitosan’s antibacterial activity against both Gram-negative (e.g., *E. coli*, *Salmonella typhimurium*) and Gram-positive bacteria (e.g., *S. aureus*, *Bacillus subtilis*). For instance, apple peel extract polyphenols enable chitosan to inhibit molds (*Colletotrichum fructicola*, *Botryosphaeria dothidea*), though yeast inhibition remains limited [60].

#### 3.2.3. Vitamin C, Titratable Acid, and Soluble Solid Content

The effects of chitosan/BLF coatings on VC, TA, and TSS content in strawberries and blueberries during storage are presented in Table 2 and Table 3.

VC is a labile nutrient highly susceptible to oxidative degradation, influenced by multiple factors such as pH, temperature, light, and the presence of enzymes, oxygen, and metallic catalysts [61]. Its retention is a critical indicator of fruit quality. For strawberries, in the control group (CK), VC content declined rapidly, decreasing by approximately 44.59% by day 8. The pure chitosan (CS) coating delayed this decline; the CS group maintained 5.77% higher VC content than CK at day 7. Among the CS/BLF treatments, the CS/BLFs-100 group demonstrated the best protective effect, retaining 6.74% more VC than CK on day 8. For blueberries, the CK group experienced rapid VC degradation, dropping to 6.44 mg/100 g by day 8 (a 42.1% loss). Chitosan coating partially mitigated VC loss (7.08 mg/100 g on day 8 for CS), while CS/BLFs-100 demonstrated the best protective effect, retaining 7.78 mg/100 g VC on day 10, which was 20.8% higher than CK (*p* < 0.01). This enhanced retention is attributed to the antioxidant activity of BLFs, which scavenge free radicals and reduce oxidative damage to ascorbic acid [47].

TA content reflecting the organic acid profile critical for flavor showed a gradual decrease in all groups during storage. For strawberries, the CK group experienced a steady decline, losing approximately 12.6% of initial TA by day 8. While CS and CS/BLF coatings marginally slowed TA loss compared to CK, the differences were generally not significant. For blueberries, TA content decreased gradually in all groups, reflecting natural metabolic processes. The CK group lost 42.5% of initial TA by day 10, while the CS and CS/BLF groups showed smaller reductions (17.8~34.6% loss). TA content showed no significant differences among the CS, CS/BLFs-50, CS/BLFs-75, and CS/BLFs-100 groups (*p* > 0.05) for both fruits, indicating a limited impact of BLFs on organic acid metabolism. This suggests that the coatings had minimal effect on inhibiting the enzymatic processes involved in acid degradation.

TSS, primarily comprising sugars and other dissolved compounds, exhibited a biphasic trend in all groups: an initial slight increase (day 0~5) followed by a gradual decrease. The early increase in CK and treated groups is likely due to water loss concentrating soluble solids [62], while the subsequent decline reflects sugar consumption during respiration [42]. CS/BLF coatings did not significantly alter the TSS trajectory compared to CK or CS, with all groups maintaining similar TSS levels throughout storage (*p* > 0.05). This indicates that adding BLFs to chitosan coatings had little influence on the TSS content, consistent with the weak effect observed for TA. The generally higher TA and TSS in coated fruits compared to CK may be attributed to the slowed respiration process caused by reduced oxygen supply from the coating film [63].

In summary, chitosan/BLF coatings effectively preserved VC content in strawberries and blueberries, likely through the antioxidant activity of BLFs, whereas their impact on titratable acid and soluble solids was negligible. These results align with the hypothesis that BLFs enhance the protective properties of chitosan primarily via antioxidant mechanisms rather than altering primary metabolic pathways related to organic acids or soluble solids [55].

### 3.3. Application of Chitosan/BLFs in the Preservation of Bamboo Shoots

#### 3.3.1. Appearance of Bamboo Shoots

**Figure 4:**
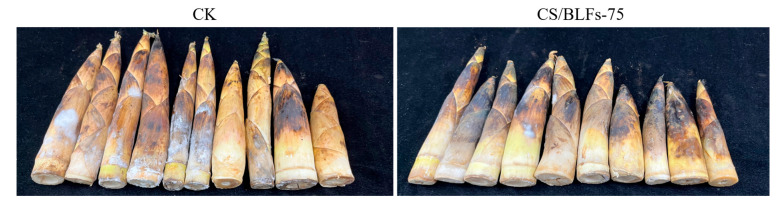
Changes in the appearance of bamboo shoots during storage.

Visual observations of bamboo shoots during storage are shown in Figure 4. By day 7, the control group (CK) displayed severe enzymatic browning, with dark brown spots on the surface and a stiff texture, indicative of rapid lignification. In contrast, chitosan/BLF-coated groups maintained significantly better appearance: the CS group showed moderate browning and slight hardening, while CS/BLFs-75 and CS/BLFs-100 groups retained lighter color and tender texture, with minimal lignification. The CS/BLFs-75 group exhibited the least surface discoloration and maintained pliability, suggesting that BLFs effectively inhibited enzymatic browning and delayed lignification, consistent with their antioxidant and antimicrobial properties.

#### 3.3.2. Weight Loss and Spoilage Percentage of Bamboo Shoots

**Figure 5:**
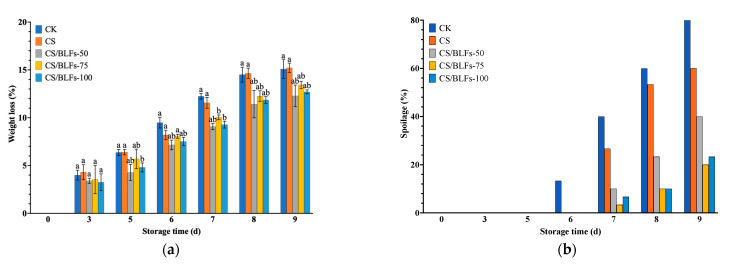
Weight loss (**a**) and spoilage percentage (**b**) of bamboo shoots stored at 25 °C for 9 days. Bars indicate the standard error of the mean. Columns labeled with different letters indicate significant differences with *p* < 0.05.

Weight loss and spoilage percentage dynamics are presented in Figure 5. The control group (CK) experienced a steady increase in weight loss, reaching 12.24% on day 7, significantly higher than all CS/BLF-coated groups (*p* < 0.01, Figure 5a). Chitosan coating (CS) reduced weight loss to 15.22% on day 9, while CS/BLF treatments further mitigated water loss: CS/BLFs-75 exhibited the lowest weight loss (13.42% on day 9), attributed to the enhanced barrier function of BLFs reinforcing the chitosan matrix.

Spoilage percentage data (Figure 5b) revealed rapid microbial growth in the CK group, reaching 83.33% by day 9, characterized by mold growth and decay. The CS group delayed spoilage onset, achieving a spoilage percentage of 60% on day 9, while CS/BLFs-75 and CS/BLFs-100 significantly suppressed spoilage, with a percentage of 20% and 23.33%, respectively. These results indicate that BLFs improve the antimicrobial activity of chitosan, effectively reducing microbial contamination on bamboo shoot surfaces.

#### 3.3.3. Vitamin C, Soluble Protein, and Crude Fiber Content

VC, soluble protein, and crude fiber content in bamboo shoots are shown in Table 4. Initial VC levels (day 0) were comparable across groups (8.77 mg/100 g). The CK group experienced rapid VC degradation, dropping to 4.22 mg/100 g by day 9 (a 51.9% loss). Chitosan coating partially mitigated VC loss (5.06 mg/100 g on day 9 for CS), while CS/BLFs-100 demonstrated better protection, retaining 5.28 mg/100 g VC on day 9 (25.1% higher than CK). This improvement is linked to BLFs scavenging free radicals and reducing oxidative damage to ascorbic acid [22]. Soluble protein content showed a gradual decline in all groups, reflecting natural protein degradation during storage. The CK group lost 21.0% of initial soluble protein by day 9, while the CS and CS/BLF groups exhibited similar reductions (9.9~12.0%). No significant differences were observed among treatment groups (*p* > 0.05), indicating a limited impact of BLFs on protein metabolism. Crude fiber content, a key indicator of lignification, increased over time in all groups. The CK group showed the steepest increase, rising from 0.95% to 1.63% by day 9. In contrast, CS/BLFs-75 and CS/BLFs-100 significantly retarded crude fiber accumulation, reaching only 1.37% and 1.33%, respectively (*p* <0.01). This suggests that chitosan/BLF coatings effectively delayed the lignification process, preserving the tender texture of bamboo shoots through the antioxidant and possibly anti-enzymatic activities (targeting enzymes like phenylalanine ammonia lyase, cinnamyl alcohol dehydrogenase, peroxidase, and polyphenol oxidase) of BLFs [9,64].

## 4. Conclusions

In this study, we developed a bio-based film system where bamboo-leaf flavonoids (BLFs) act as an effective additive for chitosan. This strategy not only enhances the tensile strength of chitosan films but also significantly reduces the need for complex functional additives in chitosan-based film preparation. Through simple physical mixing of the two components, composite films were successfully fabricated, exhibiting superior mechanical properties, functional performance, and notable free radical scavenging capacity, providing a theoretical basis for their application as bioactive packaging materials. When evaluated for food preservation, these films demonstrated excellent performance in storing strawberries, blueberries, and bamboo shoots by minimizing weight loss, inhibiting microbial growth, and preserving key nutrients, thereby effectively delaying product spoilage. Future research should focus on optimizing the chitosan/BLF film formulation for better active packaging performance and improving film stability by embedding BLFs in nano-sized carriers or grafting them onto polysaccharides.

## Figures and Tables

**Table 1:** Composition of the film-forming matrices.

Treatment	Chitosan (% *w*/*v*)	Glycerol (% *w*/*v*)	BLFs (% *w*/*v*)	Acetic Acid (1%, *w*/*v*, mL)
Control (CK)	-	-	-	-
Coating 1 (CS)	0.5	0.15	-	100
Coating 2 (CS/BLFs-25)	0.5	0.15	0.025	100
Coating 3 (CS/BLFs-50)	0.5	0.15	0.05	100
Coating 4 (CS/BLFs-75)	0.5	0.15	0.075	100
Coating 5 (CS/BLFs-100)	0.5	0.15	0.1	100
Coating 6 (CS/BLFs-200)	0.5	0.15	0.2	100

Note: CK = distilled water, CS = chitosan, CS/BLFs-25 = chitosan and 0.025% BLFs, CS/BLFs-50 = chitosan and 0.05% BLFs, CS/BLFs-75 = chitosan and 0.075% BLFs, CS/BLFs-100 = chitosan and 0.1% BLFs. CS/BLFs-200 = chitosan and 0.2% BLFs. Acetic acid (1%, *w*/*v*) was used as the solvent, with solutes (chitosan, glycerol, BLFs) dissolved in the solvent and diluted to 100 mL total volume for all treatments.

**Table 2:** Vitamin C, titratable acid, and soluble solid content of strawberries during storage.

		Vitamin C (mg/100 g)	Titratable Acid (%)	Soluble Solid (%)
Day 0	CK	54.14 ± 0.81 ^a^	0.477 ± 0.012 ^a^	10.57 ± 0.21 ^a^
	CS	54.62 ± 1.23 ^a^	0.473 ± 0.017 ^a^	10.63 ± 0.21 ^a^
	CS/BLFs-50	54.95 ± 1.41 ^a^	0.475 ± 0.007 ^a^	10.60 ± 0.10 ^a^
	CS/BLFs-75	54.80 ± 0.36 ^a^	0.478 ± 0.006 ^a^	10.60 ± 0.10 ^a^
	CS/BLFs-100	54.86 ± 0.75 ^a^	0.480 ± 0.024 ^a^	10.60 ± 0.17 ^a^
Day 4	CK	36.76 ± 0.80 ^a^	0.422 ± 0.006 ^a^	11.07 ± 0.06 ^a^
	CS	37.75 ± 1.38 ^a^	0.451 ± 0.020 ^a^	11.17 ± 0.15 ^a^
	CS/BLFs-50	38.24 ± 2.18 ^a^	0.429 ± 0.026 ^a^	11.20 ± 0.17 ^a^
	CS/BLFs-75	37.59 ± 0.29 ^a^	0.444 ± 0.029 ^a^	11.17 ± 0.32 ^a^
	CS/BLFs-100	38.94 ± 1.21 ^a^	0.435 ± 0.027 ^a^	11.23 ± 0.15 ^a^
Day 8	CK	29.45 ± 0.26 ^b^	0.417 ± 0.021 ^a^	10.20 ± 0.26 ^a^
	CS	28.28 ± 0.79 ^b^	0.445 ± 0.009 ^a^	10.30 ± 0.26 ^a^
	CS/BLFs-50	32.64 ± 2.43 ^a^	0.435 ± 0.043 ^a^	10.40 ± 0.17 ^a^
	CS/BLFs-75	30.62 ± 2.00 ^b^	0.445 ± 0.033 ^a^	10.57 ± 0.32 ^a^
	CS/BLFs-100	31.86 ± 2.04 ^a^	0.440 ± 0.019 ^a^	10.37 ± 0.15 ^a^

Note: Groups with different letters indicate significant differences with *p* < 0.05.

**Table 3:** Vitamin C, titratable acid, and soluble solid content of blueberries during storage.

		Vitamin C (mg/100 g)	Titratable Acid (%)	Soluble Solid (%)
Day 0	CK	11.12 ± 0.40 ^a^	0.377 ± 0.111 ^a^	11.10 ± 0.26 ^a^
	CS	10.90 ± 0.51 ^a^	0.506 ± 0.044 ^a^	11.17 ± 0.15 ^a^
	CS/BLFs-50	11.39 ± 0.73 ^a^	0.542 ± 0.060 ^a^	11.17 ± 0.12 ^a^
	CS/BLFs-75	11.34 ± 0.13 ^a^	0.578 ± 0.006 ^a^	11.17 ± 0.12 ^a^
	CS/BLFs-100	11.46 ± 0.44 ^a^	0.413 ± 0.073 ^a^	11.13 ± 0.23 ^a^
Day 5	CK	7.62 ± 0.38 ^a^	0.456 ± 0.152 ^a^	10.57 ± 0.06 ^a^
	CS	7.74 ± 0.10 ^a^	0.484 ± 0.133 ^a^	10.73 ± 0.15 ^a^
	CS/BLFs-50	8.00 ± 0.67 ^a^	0.362 ± 0.134 ^a^	10.77 ± 0.15 ^a^
	CS/BLFs-75	7.72 ± 0.13 ^a^	0.611 ± 0.078 ^a^	10.77 ± 0.32 ^a^
	CS/BLFs-100	8.29 ± 0.36 ^a^	0.402 ± 0.229 ^a^	10.83 ± 0.15 ^a^
Day 10	CK	6.20 ± 0.31 ^a^	0.217 ± 0.021 ^a^	9.80 ± 0.26 ^a^
	CS	6.37 ± 0.43 ^a^	0.411 ± 0.158 ^a^	9.83 ± 0.23 ^a^
	CS/BLFs-50	6.85 ± 0.84 ^a^	0.402 ± 0.145 ^a^	9.93 ± 0.21 ^a^
	CS/BLFs-75	6.71 ± 0.57 ^a^	0.378 ± 0.147 ^a^	10.13 ± 0.31 ^a^
	CS/BLFs-100	6.82 ± 0.82 ^a^	0.340 ± 0.088 ^a^	9.90 ± 0.17 ^a^

Note: Groups with different letters indicate significant differences with *p* < 0.05.

**Table 4:** Vitamin C, soluble protein, and crude fiber content of bamboo shoots during storage.

		Vitamin C (mg/100 g)	Soluble Protein (g/100 g)	Crude Fiber (%)
Day 0	CK	8.77 ± 0.70 ^a^	1.804 ± 0.121 ^a^	0.946 ± 0.033 ^a^
	CS	9.23 ± 0.14 ^a^	1.756 ± 0.193 ^a^	0.999 ± 0.001 ^a^
	CS/BLFs-50	8.61 ± 0.65 ^a^	1.828 ± 0.163 ^a^	0.963 ± 0.005 ^ab^
	CS/BLFs-75	8.33 ± 0.48 ^a^	1.932 ± 0.013 ^a^	0.987 ± 0.018 ^a^
	CS/BLFs-100	8.43 ± 0.99 ^a^	1.892 ± 0.050 ^a^	0.976 ± 0.030 ^a^
Day 5	CK	6.11 ± 0.54 ^a^	1.657 ± 0.106 ^a^	1.251 ± 0.033 ^a^
	CS	6.11 ± 0.40 ^a^	1.692 ± 0.166 ^a^	1.196 ± 0.018 ^a^
	CS/BLFs-50	5.74 ± 0.86 ^a^	1.759 ± 0.155 ^a^	1.070 ± 0.204 ^a^
	CS/BLFs-75	6.03 ± 0.31 ^a^	1.831 ± 0.028 ^a^	1.048 ± 0.029 ^b^
	CS/BLFs-100	6.56 ± 0.78 ^a^	1.785 ± 0.091 ^a^	1.047 ± 0.202 ^a^
Day 9	CK	4.22 ± 0.69 ^a^	1.425 ± 0.093 ^a^	1.634 ± 0.026 ^a^
	CS	5.06 ± 0.26 ^a^	1.553 ± 0.165 ^a^	1.398 ± 0.019 ^b^
	CS/BLFs-50	5.68 ± 0.78 ^a^	1.611 ± 0.137 ^a^	1.360 ± 0.024 ^b^
	CS/BLFs-75	4.65 ± 0.29 ^a^	1.741 ± 0.022 ^a^	1.372 ± 0.017 ^b^
	CS/BLFs-100	5.28 ± 0.36 ^a^	1.665 ± 0.028 ^a^	1.328 ± 0.046 ^b^

Note: Groups with different letters indicate significant differences with *p* < 0.05.

## Data Availability

All the data of this research are included in this manuscript.
